# A qualitative process evaluation of community-based participatory research and human-centered design in the ‘Let’s talk about vaccines’ approach in Mozambique and Malawi

**DOI:** 10.1186/s40900-025-00677-4

**Published:** 2025-02-21

**Authors:** Linda Shuro, Emily Lawrence, Jeroen De Man, Lucia Knight, Helen Schneider, Hanani Tabana

**Affiliations:** 1 School of Public Health, University of the Western Cape, Robert Sobukwe Rd, Bellville, Cape Town, 7535 South Africa; 2 VillageReach, 210 S Hudson St, Suite 307 Seattle, Washington, 98134 USA; 3 School of Public Health and Family Medicine, Faculty of Health Sciences, University of Cape Town Falmouth Building, Anzio Road, Observatory, Cape Town, 7925 South Africa; 4 School of Public Health and SAMRC Health Services to Systems Research Unit, Faculty of Community and Health Sciences, University of the Western Cape, Cape Town, South Africa

**Keywords:** Community-based participatory research, Human-centered design, Under two immunization, Low and middle-income countries, Qualitative process evaluation, RE-AIM, Consolidated framework for implementation research

## Abstract

**Background:**

Ensuring full coverage of childhood vaccination programmes is a persistent challenge in low- and middle-income countries. Urgent action is required to ensure catch up of missed immunisations in children, while simultaneously building trust and demand within communities to sustainably address existing immunization gaps. This paper summarizes the findings of a process evaluation of the ‘Let’s talk about vaccines’ approach by VillageReach in Mozambique and Malawi. The approach used community-based participatory research to identify the barriers to childhood vaccination faced by caregivers and healthcare workers, with human-centered design to codesign potential interventions to improve under two immunization access and uptake.

**Methods:**

To evaluate the implementation of the ‘Let’s talk about vaccines’ approach we conducted a qualitative process evaluation guided by the Reach Effectiveness Adoption Implementation Maintenance framework and Consolidated Framework for Implementation Research (CFIR). We completed a total of 76 qualitative interviews and 85 self-administered surveys among caregivers, healthcare workers, health officials and other stakeholders involved in the approach. We transcribed the interviews verbatim and analysed them using thematic analysis and constructs of the RE-AIM and CFIR frameworks. We analysed the survey results in Excel.

**Results:**

Key elements of the approach contributing to high fidelity to community-based participatory research principles in both countries, included diverse collaborative study and project teams, involvement of eight caregiver researchers, novel and traditional participatory methods, and extensive mobilization efforts. Success factors for human-centered design in the ideation and prototyping phase included fostering equal participation and empathy, value placed on each participant’s input, mitigating inherent power differences, interactive feedback processes, and extensive iterative processes leading to tangible solutions. Challenges included adjusting to new methods and contextual realities. Factors influencing the potential adoption of the approach included locally developed solutions, participant involvement, collaboration, a major advantage over alternative approaches, ease of use of the co-created interventions, alignment with government objectives, and adaptability for system-wide integration into immunization programming. The potential sustainability of the approach was supported by the involvement of health ministries, health professionals, community representatives, and capacity building of local structures. However, resource and incentive constraints posed as a potential challenge to maintaining long-term motivation and action.

**Conclusion:**

The evaluation findings from the ‘Let’s talk about vaccines’ approach highlighted key elements for applying community-based participatory research and human-centered design to collaboratively identify immunization barriers and create tangible solutions to overcome them. By integrating these approaches into routine immunization programs, it can potentially improve vaccination efforts for children under two in low and middle-income countries, leading to lasting change. Supporting policies that prioritize community involvement in research, program design and implementation and sustainable funding enhances immunization strategies, ensuring that they are tailored to local needs.

## Abstract (plain language summary)

Childhood vaccinations are important for protecting children worldwide from serious diseases such as measles, diphtheria, and tetanus. However, many children have missed out on important vaccines because of several challenges, especially in low- and middle-income countries. Our evaluation focuses on understanding how participatory activities (community-based participatory research and human-centered design) conducted by VillageReach in several communities in both Mozambique and Malawi included communities in identifying challenges faced in immunizing children under two and developing solutions with caregivers, health providers, and other relevant individuals involved in immunization planning. We interviewed these participants, exploring how the activities were carried out. Soon after the workshops, we asked participants to fill out questionnaires to get immediate feedback. The evaluation results showed what works well in the approach, what challenges exist, as well as what can help and hinder the successful use of these participatory activities. By closely following community-based participatory research and human-centered design, the approach was implemented with success. In this paper, we share lessons from the approach that can help improve participation in childhood immunization efforts in similar communities, aiming to close the gap and reach more children with necessary vaccines.

## Background

Childhood vaccinations stand as one of the most cost-effective public health interventions, yet global coverage has faltered, with 67 million children missing out on routine immunization [1, 2]. Building trust and demand while addressing immunization gaps within communities has become a core objective of global agencies such as the United Nations Children's Fund (UNICEF), Global Alliance for Vaccines and Immunization (GAVI), and the World Health Organization (WHO) [1]. The importance of these factors was recently emphasized during the COVID-19 pandemic. The COVID-19 pandemic exacerbated existing issues such as mistrust in the healthcare system [2] and a lack of vaccine uptake, resulting in a concerning increase in the number of children who missed vaccination. To achieve trust and demand, the involvement of communities (including the intended target population) is essential. Community-based participatory research (CBPR) and human-centered design (HCD) are emerging as promising strategies that involve communities collaboratively to improve health outcomes and foster trust in healthcare services [3–6]. These methodologies focus on inclusive, collaborative strategies that involve community members and stakeholders in every stage of the research and implementation processes. CBPR involves researchers and community stakeholders in the research process with shared power, recognizes experiential understanding, and focuses on improvements in circumstances and implementation [7, 8]. CBPR is also cited as an approach that incorporates education and social action to improve health and social outcomes [9]. To address health and socioeconomic disparities, CBPR, situated within participatory research methodology, places a strong emphasis on community involvement in study design, implementation, and dissemination. HCD integrates an inclusive participatory process that results in the collaborative development of solutions to problems, buy-in by stakeholders, and better-tailored solutions [5]. It is an approach that centers on creativity in an iterative process to bring human-centered views to the development of feasible solutions. It consists of three main phases: inspiration, ideation, and implementation [10]. Within the framework of design thinking approaches, HCD emphasizes creativity, user empathy, and iterative problem-solving to create solutions that are tailored to the individual needs of users [11].

The use of these approaches in improving public health interventions, such as routine immunization, offers a paradigm shift away from how solutions to enhance vaccination coverage have traditionally been developed by international stakeholders and national governments, overlooking the insights of healthcare workers and caregivers in identifying barriers and solutions [11–16]. There is a dearth of studies, especially in low- and middle-income countries (LMICs), that provide evidence on the cocreation of interventions using CBPR and HCD, particularly within the immunization space [16, 17]. Limited evidence exists concerning how these approaches may firstly lead to improved design, adoption, and effectiveness of interventions [6, 17] and secondly, how they complement each other in practice to solve public health challenges [4]. The literature shows the use of traditional mixed methods approaches such as surveys, interviews, and ecological studies in the identification of drivers of dropouts, with few studies using participatory approaches [18, 19].

To address the existing evidence gap, we conducted a qualitative process evaluation of the implementation process of the ‘Let’s talk about vaccines’ approach (Bate Papo in Portuguese), which combines CBPR and HCD to identify and address barriers contributing to immunization dropouts among children under two in select districts of Mozambique and Malawi [20, 21]. Process evaluations assist in evaluating the process of implementation, identifying barriers and facilitators to implementation of interventions [22]. The evaluation draws on insights from the intended target population and health providers involved in immunization services and programming. The purpose of the evaluation was to assess the implementation of community-based participatory research and human-centered design in engaging local communities to explore and address drivers of immunization among children under two years old.

The objectives of the process evaluation were;

- To determine the extent to which the approach has successfully engaged local communities in identifying factors affecting under-two immunization and in co-creating potential solutions; and
- To identify opportunities and challenges in involving communities in discussions and actions related to childhood immunization.

### Description of the CBPR and HCD Approach of the ‘Let’s talk about vaccines’ project under evaluation

‘Let’s talk about vaccines’ project was developed and implemented by VillageReach, a global not-for-profit organization working to improve access to quality healthcare in the world’s most under-reached communities. The project aims to understand the barriers that caregivers face when trying to fully vaccinate their children and to design, implement, and evaluate community-driven solutions to reduce the problem of routine immunization dropouts in selected, hard-to-reach areas in various districts in Mozambique and Malawi [20, 21]. The project design used the principles of CBPR and HCD (Let’s talk about vaccines approach) to engage community members and healthcare workers to generate new knowledge and targeted solutions that meet their needs in three primary ways:

1. Caregiver training: Local caregivers, defined as women who had primary responsibility for caring for a child under the age of two, from the community were trained in photovoice and photo elicitation to conduct qualitative research.
2. Data collection: Caregivers, healthcare workers, health officials including Expanded Programme on Immunization (EPI) officials, and community leaders collected and reviewed qualitative data to identify barriers and brainstorm potential solutions.
3. Cocreation: Caregivers and healthcare workers co-created solutions that work for them and participate in solution implementation.

The ‘Let’s talk about vaccines’ approach phases included the following:

Phase 1- Identify: Eight Caregiver researchers, four from each country, were hired, trained in CBPR methods and analysis and became part of the project team. Caregiver researchers were central to driving the data collection and analysis process to explore the reasons for vaccination dropout from caregivers and healthcare workers. By applying the WHO’s Behavioral and Social Drivers of Immunization Model (BeSD) [23, 24], the project team identified key themes across study sites in both countries, identifying common barriers in routine immunization dropout across different contexts.

Phase 2- Implement: Using principles of HCD during the ideation and prototyping phases, unique community-identified interventions were codesigned in each country to reach under-immunized children. During the ideation phase, participants engaged in creative and collaborative sessions including brainstorming, personas, power-balancing activities, and role play to generate, prioritize, and vote on potential solutions, drawing on the insights from caregivers, healthcare workers, and stakeholders involved in immunization programming and influencing uptake. Following the ideation phase, the identified solutions (prototypes) were finalized for selection with the EPI and further developed through an iterative prototyping process with participants from Phase 1 and EPI officials into tangible intervention components.

This paper presents the evaluation findings of this approach as part of a qualitative process evaluation.

## Methods

### Study design

We conducted a qualitative process evaluation to explore the implementation of the ‘Let’s talk about vaccines’ approach from various stakeholder perspectives in engaging communities to identify drivers and co-create solutions for immunization for children under two. We used the RE-AIM framework, along with the Consolidated Framework for Implementation Research (CFIR) to guide our evaluation.

### Theoretical frameworks applied in the evaluation: RE-AIM and CFIR

The RE-AIM framework offers a structured approach for evaluating interventions, focusing on reach, effectiveness, adoption, implementation, and maintenance dimensions. It has been widely utilized in low- and middle-income settings providing clear and identifiable outcomes that indicate the extent and impact of the intervention [25–27]. The CFIR framework is an explanatory framework detailing modifiable factors that influence implementation across different contexts, explaining why the implementation was successful or not [28]. It comprises 39 constructs organized into five domains: Intervention characteristic; Outer setting; Inner setting; Characteristics of individuals; and Process [27, 29]. In this evaluation, we applied the implementation, adoption, maintenance, and effectiveness dimensions from the RE-AIM framework and relevant constructs from three domains of CFIR (intervention characteristics, inner setting, and outer setting). The CFIR constructs were aligned with the RE-AIM adoption dimension to gain further insight into the factors influencing the potential adoption of the approach (see Table 1). This combination resulted in a tailored conceptual framework for our qualitative process evaluation [30, 31] with these dimensions and constructs guiding both the data collection tools and the analysis of the findings.

**Table 1:** RE-AIM/CFIR and evaluation focus

RE-AIM dimension	Evaluation focus
Implementation (fidelity)	Perspectives of stakeholders on VillageReach’s approach adhering to the CBPR and HCD process and any adaptations (adjustments made during implementation)
Effectiveness (qualitative evidence)	Perceptions of the benefit of the approach in immunization planning and knowledge, while also considering unintended consequences
Adoption	Perspectives of caregivers and health service providers on acceptability and uptake of the approach in both country contexts
	CFIR Domains and related constructs *Intervention characteristics domain*: Intervention source—Perception of key stakeholders about whether the intervention is externally or internally developed Evidence strength, and quality—Stakeholders’ perceptions of the quality and validity of evidence supporting the belief that the intervention will have desired outcomes Relative advantage- Stakeholders’ perception of the advantage of implementing the intervention versus an alternative solution Complexity—How straightforward or complicated the intervention is to use *Inner setting domain:* Networks and communication—how information flows, interactions occur, and relationships are formed, collaborative decision making Implementation climate—acknowledgment of efforts and contributions by individuals *Outer setting domain*: External policies and incentives—external factors that can facilitate or hinder the implementation of an intervention
Maintenance	Perspectives on the sustainability or maintenance of the approach

Table 1 below highlights the applied RE-AIM dimensions, CFIR domains and evaluation focus.

### Study setting and participant recruitment and selection

This evaluation was conducted at the VillageReach study sites in the Namarroi and Gile districts located in Zambezia Province, Mozambique, and in the Lilongwe and Mzimba North districts in Malawi. Gile and Namarroi are rural districts where most communities are located far from health facilities. Lilongwe is an urban district, and Mzimba North is rural. We recruited participants involved in the approach from the eight ‘Let’s talk about vaccines’ sites in Mozambique and eight sites in Malawi. Each site consisted of a health center and its associated catchment area.

### Sampling

Working closely with VillageReach project staff, we used purposive sampling to select participants who were involved in all or some of the phases of the ‘Let’s talk about vaccines’ approach in both countries [32]. In Mozambique, we conducted 45 individual interviews between June and November 2022. In Malawi, we interviewed 31 participants between February and August 2023. Participants included caregiver researchers, caregivers, healthcare workers, community actors, health officials including EPI representatives, and VillageReach staff. At least 1 individual from each participant category was included in the study, except for the caregiver researcher’s category in Namarroi, Mozambique, who were no longer involved in the project at the time of the evaluation. Further details regarding the breakdown of participant categories can be found in Appendix 1.

### Data collection

Data collection consisted of semi-structured interviews to obtain insights and administering self-completed surveys to obtain immediate feedback from stakeholders engaged in the approach. We conducted both interviews and surveys concurrently with the implementation of the approach. The evaluation team, consisted of a researcher, two research assistants, and two translators. As part of the preparation for the interviews, we reviewed relevant project documents and conducted an audit trail of activities for each country [33]. Additionally, we observed training sessions for the intervention components in both Mozambique and Malawi and participated in virtual data collection briefs to gain further insights into the process of implementation.

#### Semi-structured interviews

We conducted a total of 76 semi-structured interviews with the selected participants from Mozambique (45) and Malawi [31], which lasted approximately 45–60 min using semi-structured guides that we developed based on the RE-AIM framework and tailored to each participant category. The interviews aimed to gather information about participants’ involvement in the project; their perceptions of the CBPR and HCD process; their outcomes and sustainability, strengths and weaknesses; and their recommendations for improving future CBPR and HCD processes. We conducted interviews in Portuguese and Lomwe at the district and provincial levels in Mozambique and in English, Chichewa, and Tumbuka in Malawi. Prior to participation, we obtained informed consent from all respondents.

#### Participant feedback survey

We self-administered a questionnaire adapted from Bartlett et al. [6] based on the principles of HCD to various participants including caregivers, healthcare workers, community health workers, community leaders, and district health officials to collect immediate feedback after the HCD workshops concluded. In June 2022, 15 participants in Mozambique provided feedback during the HCD prototype workshop. In February 2023, we distributed feedback surveys to 19 participants in Lilongwe and 20 in Mzimba North during the HCD ideation workshops. Additionally, the prototype workshop in Malawi in June 2023 involved 31 participants who provided their feedback.

### Data analysis

The interviews were transcribed verbatim by two researchers from Mozambique and Malawi, and we checked them to ensure the reliability of the data. We imported the qualitative data into ATLAS.ti 22, and analysed it. The analysis used a combination of framework and thematic analysis with both inductive and deductive components[26, 27, 29, 30, 34]. Initially, coding commenced as an inductive process to become familiar with the extensive dataset and emerging concepts and codes. The deductive coding of the transcripts was subsequently guided by the RE-AIM dimensions, 9 CBPR principles, HCD principles [35], the approach's intended goals, and relevant CFIR domains to structure the data. We then charted the data by organizing the relevant text and codes into thematic categories according to the frameworks. Thereafter, we summarized and streamlined the text to identify patterns and key insights aligned with the RE-AIM dimensions, CFIR domains, and the CBPR and HCD principles. This process aligns with the Critical Appraisal Skills Programme (CASP) guidelines for conducting rigorous qualitative research [36]. We imported the survey results from the self-administered questionnaires into Excel and analysed them to assess the frequency of responses for each question. We then created frequency charts to visually represent the category of participants and represent results. We summarized key findings and integrated them into a report to convey the survey results.

### Trustworthiness

The inclusion of an extensive number of participants from both countries in this evaluation contributed to its credibility. We presented the preliminary findings to the project team in both countries for validation, allowing for checks on interpretations, and their consistency with participants’ experiences. Additionally, we triangulated data sources such as interviews, participant observations, and surveys for a detailed set of results and provided a thick description of the findings. An audit trail detailing how the data was collected, analysed, and interpreted established conformability. For reliability, we kept all the raw data from each interview. Furthermore, the description of the study setting, and methods provides information that enhances transferability to similar contexts.

### Ethics approval and statement

This evaluation involved human participants and was approved by the Biomedical Science Research Ethics Committee of the University of the Western Cape (Ethics Reference Number: BM22/4/3), the Mozambique National Bioethics Committee for Health (Reference: 588/CNBS/22), and the National Health Sciences Research Committee of Malawi (Protocol # 22/08/2987) (see additional file 1). The study adhered closely to the ethical guidelines and regulations of the respective ethics committees. Informed consent was obtained from all participants, and anonymity and confidentiality were maintained throughout the study. Participation in this evaluation did not result in any negative consequences.

### Limitations of the evaluation

The evaluation had a multi-site component, which introduced factors such as differing country ethics approval, ongoing polio campaigns, and instances of flooding. As a result, maintaining precise alignment with the iterative fast-paced nature of the approach proved challenging. However, we were able to adapt and engage in most of the processes involved. Additionally, there may be some form of bias in the evaluation, as participants’ responses may be slightly altered as a reaction to an evaluation of processes that they were part of and responsible for. While the feedback survey findings were complementary to the qualitative findings, it must be noted that the smaller sample size presents a limitation for broader statistical generalizations.

## Results

The results of this qualitative process evaluation were structured according to specific process dimensions and domains drawn from the RE-AIM and CFIR frameworks (see Table 1).

### Implementation

#### Key elements in implementation

Respondents in Mozambique and Malawi noted key elements in the implementation of the CBPR study and codesign (HCD) phase, demonstrating high fidelity to the CBPR and HCD principles. These included collaborative study teams, caregiver researcher involvement, inclusive participatory methods, and extensive iterative processes. Below are the evaluation findings for each phase of the approach:

#### Phase 1: CBPR study

According to the participants involved in the study, one of the key elements of the CBPR was VillageReach’s establishment of diverse collaborative core structures such as study and project teams in each country. These teams included individuals who were familiar with the context and affected by issues. Members included project team staff, caregivers, in-country co-investigators, and health officials. A health official from Mozambique elaborated:

*“There was a study group that was doing research, which first involved the community itself: the caregivers, all the influencers in the community who knew the caregiver, and the whole family. All the health professionals who carry out immunization activities in the health facilities were involved, the Directorate, including the District Director and the Medical chief, the administrator, the heads of the localities, the heads of the posts, the VR [VillageReach] colleagues, and the person responsible for the EPI.”* (Health Official, Mozambique)

Respondents highlighted a noteworthy achievement of establishing well-organized community structures in the CBPR, involving community health workers; leaders from various sectors (such as community, religion, and education), caregivers and community members. These structures served as platforms for fostering collaboration and improved the coordination of under two immunization efforts among stakeholders at different levels. By actively engaging these structures, the project adopted a bottom-up approach, facilitating positive relationships among all involved actors. Health officials noted:

*“The strong point is involvement. If the message for vaccination is delivered through a community leader or focal point or someone who lives in the same community, it is more comprehensive than the message delivered by a health worker. We speak in Portuguese, and they speak the local language, so letting them spread the message is more positive…. We train the community leaders to mobilize the community, and they manage to be more assertive and mobilize the population.”* (Healthcare worker, Mozambique).

*“I really appreciated the contribution that we get from the community and other stakeholders… Sometimes it could have been tilted towards what I think as an office, top-down approach, but in this case, it was a down-top approach…the bottom-up approach has revealed the real issues from the ground that can solve the problems or a low uptake of immunization in these respective areas that the project is in.”* (Health official, Malawi).

Another important element cited was the value of caregiver-led research, which involved eight caregiver researchers, who actively engaged as part of the project team. They were at the center of driving the data collection process, prioritizing the voices of caregivers. Furthermore, their ability to speak the local language helped improve participation from caregivers and the community, enhancing the project’s reach and fostering a sense of ownership and collaboration within the project team. This approach helped to build trust within the communities involved in the project. A caregiver researcher from Malawi and Mozambique shared their experiences:

*“I underwent a one-week kind of training in Mponela whereby we were formally introduced to the study but also what we were expected to do. But also, by the end of that as well we had a pilot…also we were introduced to some of the forms, for example, the data collection forms, the photos, and everything.”* (Caregiver Researcher, Malawi)

*“As I collected the data, I consider that my involvement was direct. In addition, while collecting the data and making the transcripts, we were able to make a general analysis and in this way be able to report to the health facility what happens in the community.”* (Caregiver Researcher, Mozambique)

Another key implementation element was the mix of novel and traditional participatory methods, such as photo voice in Mozambique and photo-elicitation in Malawi with caregivers, along with electronic messaging and interviews with healthcare workers. Respondents explained that the process had encouraged active engagement and out-of-the-box thinking, capturing diverse experiences and viewpoints regarding access to and uptake of under two immunizations. It had successfully amplified the voices of caregivers and healthcare workers, who are directly affected and involved in immunization efforts. For example, caregivers and healthcare workers expressed their involvement in this process:

*“At first, when photo voice appeared, mothers had the freedom and opportunity to explain their experiences based on their own image, so it was very relevant. The method itself was very interesting and managed to cover both educated and non-educated mothers.''* (Caregiver researcher, Mozambique)

*“Yes, we received some training and also, we received some cameras to take pictures of the children, in order to show the phases of the children after vaccination.”* (Caregiver, Mozambique)

*“So, during the telegram exchanges, we were asking them [healthcare workers] through some of the things that we saw during the observations at the health facilities... and then after those messages, we had a follow-up interview where it was also …open-ended questions…we were trying to learn about some of the things that we observed during the observations and …through the SMS messages...”* (Caregiver researcher, Malawi)

“*They [VillageReach] reach[ed] the right people for them to know the barriers that prevent children from being fully vaccinated; health workers and caregivers both had challenges, and they found solutions to themselves.”* (Health Surveillance Assistant, Malawi)

#### Challenges and adaptations

Some challenges which emerged included caregivers’ initial hesitation with photovoice and photo-elicitation as well as healthcare workers’ concern about data confidentiality. In Mozambique, caregivers were hesitant to use cameras and share images, whereas healthcare workers were worried about the potential repercussions of sharing information. Similarly, in Malawi, some caregivers found it challenging to relate to photos taken in rural settings in Mozambique, which were used in an urban context. Adjustments, such as capturing locally relevant images, and providing additional orientation on the study to healthcare workers helped address these issues.

In Mozambique, caregiver researchers initially encountered challenges with questioning techniques during the first interviews and the new photovoice method, resulting in a temporary pause in data collection. To overcome these challenges, the project team organized exercises to improve the questioning skills, leading to improved questioning in subsequent interviews. Weekly virtual debriefing sessions were conducted that facilitated mutual learning among the team, providing valuable feedback and training where needed. A health official from Mozambique, a caregiver researcher from Malawi, and project staff, provided insights into these challenges:

*“In the first phase, the challenges were the fear of mothers with their images, that these could be disclosed and also at some point, the health professionals understood that they would be harmed, the information of a mother would come out to report some information to incriminate the health professional, but these challenges were eliminated through an explanation to mothers and health professionals that the study is not intended to hurt anyone, but rather to bring solutions.”* (Health Official, Mozambique)

*“…we felt like some of those photos were not representing our situation. I think the difference, …was that most of the photos were from a rural setting…. So, we had to ask… if we could have another set of photos … to take some urban photos which had some real situations that happened in the areas that we were working in.”* (Caregiver researcher, Malawi)

*“…we did like a whole data pause and like some exercises, where we had the caregiver researchers review their transcripts and like identify where they had asked leading questions where they could approach more… interviews improved dramatically after that exercise…”* (VillageReach).

#### Phase 2: Codesign phase (HCD)

During the co-design phase, which included ideation and prototyping, participants in both countries offered insights into facilitators and areas for improvement, as explained below.

### Ideation

In the ideation workshops, various participatory activities such as brainstorming, engaging with personas, voting on possible solutions, group discussions, and role-playing were used, with participants acknowledging the value that had been placed on each participant’s input and the efforts made to balance power dynamics. This created an equal platform for participation and mitigated inherent power differences among participants. Participants noted that the CBPR study findings were presented during these workshops and that they actively engaged in interactive feedback processes. These activities allowed participants to explore contextual barriers, validate study findings, and freely express their perspectives within the process. As a result, there was a strong sense of ownership and buy-in to the approach. A health official and health surveillance assistant (HSA) described their experiences with the process:

*“There was first the presentation of the problems encountered, we asked for possible solutions, and in a third phase, the members/participants voted on the solutions. We exposed the problems on the wall, and we all went to visit those problems… each one would vote on the most relevant problems and then we brought the possible solutions.”* (Health official, Mozambique)

*“At first, we were discussing in groups, after discussing in groups that is when now we started brainstorming and prioritizing these ideas, and we could identify strong ideas and paste them separately, we were pasting only those ideas we feel like they would help us.”* (HSA, Malawi)

A community volunteer from Malawi reflected on power dynamics during the workshops:

*“We were all equal; no one was powerful. Everyone was able to share his/her thoughts. We were able to enlighten one another; we agreed on one thing.”* (Community Volunteer, Malawi)

### Prototyping

The prototype phase in both countries involved an extensive iterative process. This included workshops with caregivers and stakeholders, consultations with health officials and health promotion experts, design workshops for message development, and multiple rounds of pre-testing solutions with several iterations. Participants shared that this process led to tangible, context-specific solutions that resonated with their lived realities. District-level discussions with various stakeholders helped refine solutions, ensuring they were suitable for each district’s context. A health official described the process as follows:

*In the two districts, there were discussions between colleagues from the other health facilities... Then these colleagues went to the health facility where they met with the APSs [Agentes Polivalentes de Saúde-Community Health Workers] about prototyping. Afterward, there was a meeting between the two districts in Mocuba... In that meeting, each health facility brought an APS and a more influential community leader…to harmonize ideas/solutions from one district with another in order to obtain a unique experience.”* (Health official, Mozambique)

In Mozambique, co-created interventions included implementing immunization education sessions to enhance caregiver knowledge and agency, prioritizing mobile brigades to improve access to immunization services for hard-to-reach populations, and establishing monthly collaborative immunization planning sessions to enhance coordination and communication between health facilities and communities. In Malawi, co-created interventions included the use of Amplio Talking Books [37] and drama performances to disseminate essential immunization information, enhance community involvement, and implementing a community-based scorecards process to foster community engagement and accountability. Participants confirmed through interviews and feedback surveys (Appendix 2) that their input was visibly integrated in the final solutions as reflected in the solution training. Representatives from the mother care group and drama group in Malawi described the participatory nature and extensiveness of the process in one of the prototype workshops:

*“After meeting up with the group from Lilongwe, we were put into groups. These groups were mixed up, where both and individuals from both Lilongwe and Mzimba North were found in each group to discuss. It was found that problems presented by the groups from Lilongwe and Mzimba were similar.”* (Mother Care Group Representative, Malawi)

*“We were put in groups trying out things related to drama and vaccines. There was a lot we were taught related to vaccines.”* (Drama Group Representative, Malawi)

A review of VillageReach HCD workshop reports highlighted that brainstorming potential solution ideas and prototyping generated the most interactions among participants, with more time spent discussing each idea. However, this also meant that equal time was not given to discuss all the suggested ideas. Feedback from the respondents revealed the project’s success in cultivating a deep sense of empathy and fostering self-reflection among healthcare workers and EPI officials. Through HCD activities such as persona reflection, participants were encouraged to reflect on the vaccination process, identifying areas where improvements could be made, and leading to deeper interactions with caregivers. This, in turn, developed a better understanding of the realities faced by caregivers. Healthcare workers reflected:

*“… despite being a health professional, I did not understand why some mothers are unable to complete the vaccination of their children. We know that there are some taboos that the community had that didn’t even reach us. Then I also realized that some mothers didn't complete the vaccines not because they didn't want to, but the circumstances in which they live don't make it easy. At some point, the behavior of health professionals, that also forces mothers to give up coming.”* (Health care worker, Mozambique).

*"My opinion is that, this program will help and it has made us health workers, and as someone who looks at the issues of vaccines, it has reminded me some things through interviews and questions that we have been asked frequently because there are some issues that we do usually, but when we have questions or interviews, you are reminded some of the things that you were not doing to start doing them."* (HSA, Malawi).

#### Challenges and adaptations

The term “prototyping”, initially challenging for participants as a new concept, evolved into a more receptive idea when reframed as “solution presentations”.

#### Summary of the alignment of the approach to CBPR principles

A summary of a qualitative assessment presented in Appendix 3 demonstrates that the approach substantially aligns with CBPR principles.

### Adoption of the approach and co-created interventions

In both countries, feedback from respondents on the potential adoption of the project revealed factors influencing the potential adoption of the approach and co-created interventions. These factors are summarized in Table 2 below, according to the CFIR domains and constructs. Supporting quotes are available in Appendix 4.

**Table 2:** Factors influencing the potential adoption of the approach

Adoption	
CFIR Domain	CFIR Construct and factors explained
Intervention characteristics domain	Intervention source: locally developed solutions Despite initial external global and regional consultations (i.e., the use of theatre-based methods and reducing dropouts), participants appreciated the locally developed and feasible solutions tailored to their contexts
	Evidence strength and quality: alignment of study findings with community realities Respondents validated CBPR study findings in HCD ideation workshops and meetings with provincial and district health officials, affirming alignment by connecting findings to their own experiences and community realities
	Relative advantage: approach seen as a major advantage compared to alternatives Participants recognized the introduction of novel methods, such as involving caregiver researchers and utilizing photovoice and HCD, as fostering community participation. These innovative methods were seen as a major advantage compared to alternatives
	Complexity: ease of use of the co-created interventions In both countries, the potential ease of use of the co-created interventions is a facilitator for the potential adoption of the project interventions. Interventions were perceived as user-friendly and could easily be used by existing community health structures, such as APSs in Mozambique and community volunteers and Mother care group representatives in Malawi
Inner setting domain	Effective networks and communication: collaborative initiatives and shared decision-making process Various collaborative initiatives such as training workshops, regular meetings, diverse study teams, community structures and communication platforms such as virtual debriefs, and WhatsApp exchanges were implemented which fostered effective networks improving communication and collaboration in immunization programming. Participants highlighted the shared decision-making process, which resulted in consensus on identified problems and solutions, despite some geographical differences. This collaborative approach allowed for interventions to be relevant and applicable across different settings
	Implementation climate: collective openness to ideas and adaptability of interventions Respondents appreciated that their ideas and contributions were highly valued and reflected in the final interventions, fostering ownership and commitment to the project and further enhancing their buy-in to the project and its outcomes. Participants noted that certain components such as incorporating the caregiver researcher role at the community level, providing HCD training to the Ministry of Health/EPI officials, and enhancing mobilization and vaccination awareness efforts were adaptable for broader immunization programming. These components were perceived as suitable for addressing community-level challenges and beyond. However, system-level challenges such as lack of transport and funding were noted as persisting barriers
Outer setting domain	External policies and incentives: alignment with government objectives The project was widely recognized for its alignment with government objectives and was welcomed at the district level. Participants expressed confidence in the interventions’ ability to improve immunization indicators, increase vaccination coverage, and garner strong ownership and buy-in of the process. The capacity-building aspect was acknowledged for its positive impact, particularly in engaging caregivers and improving community involvement in immunization services

## Maintenance

While sustainability may extend beyond the project's designated timeframe, certain aspects were observed that could potentially support or affect it. These aspects include the active involvement of ministries of health at various levels and stakeholders in CBPR and HCD workshops in both countries. The project focused on building capacity within existing health and community structures, equipping participants with intangible skills to continue mobilizing caregivers and identify barriers and potential solutions. For example, one caregiver researcher shared a tangible example of how the project’s workshops prompted health officials to initiate community projects independently:

*“I am really hoping this thing [approach] is going to be sustainable even beyond Bate Papo because they are things that came from them and they have seen the gap within themselves. I would even give an example, the Senior HSA [Community Health Worker] was telling me the other day you know…after participating in those workshops, then the whole brainstorming, the whole coming up with ideas and what really opened our mind and when we come back …and sat down as a team to say, ‘What is it that we can do? We are really lacking in our community.’ Then they…had a meeting with one of their communities and until they had started the process of moulding the bricks to have a shelter for their outreach but all along the outreach has been there, [but they] didn’t feel the need of having a shed.”* (Caregiver researcher, Malawi)

Perceived resources and time intensity required for the approach posed concerns regarding sustaining motivation and action, particularly among community actors, such as community health workers and community volunteers, due to resource constraints within the health systems of both countries. A health official in Mozambique noted this challenge: *“What may fail in the future, for example, the question of incentives, they are not permanent, at some point they will end so the activities will fail because there will be no such meetings, there will be no motivation.”* (Health official, Mozambique).

## Discussion

This evaluation provides insights into how the ‘Let's talk about vaccines’ approach demonstrates the potential of using CBPR and HCD strategies to engage communities in participatory data collection processes, identifying the drivers for under two immunization and collaboratively bringing human-centered views in designing solutions. It also highlights the challenges and opportunities in engaging communities in childhood immunization issues. Using the CBPR and HCD approaches alongside the BeSD model [23] for analysis provided a comprehensive and inclusive framework to identify key behavioral and social drivers influencing immunization dropouts. This approach allowed for the development of tailored interventions that could potentially address the underlying reasons for non-compliance with immunization schedules. Emphasizing a bottom-up approach, caregivers, healthcare workers, community members, and stakeholders collaboratively contributed, fostering ownership and sustainability [38, 39].

Interventions aimed at improving intangible elements, when co-designed with the target population and stakeholders, contextually adapted, and implemented in tandem with structural improvements, hold the potential to drive sustainable changes in immunization efforts and health systems at large [40]. Within resource-constrained contexts, the project’s capacity to "rewire" intangible aspects of immunization efforts serves as a crucial foundation for its success. This rewiring involved fostering collaborative partnerships, building trust and empathy among communities and health providers, promoting motivated and participatory leadership, and facilitating cross-sector coordination. Ramani et al. [40] highlighted that these intangible elements are often overlooked, but their study in India demonstrated that approaches aimed at breaking down power and gender hierarchies and enabling collaborative work across different cadres show promising results in achieving equitable health service delivery. Transdisciplinary teams, stakeholder training, and the application of flexible, inclusive HCD methods in the ‘Let’s talk about vaccines’ approach were pivotal in addressing power dynamics and fostering empathy during the co-design process, ultimately leading to practical and tailored solutions. Critically, this approach helped reveal vulnerabilities within the health system. Systemic challenges faced by health service providers, such as resource constraints, vaccine stockouts, and a lack of transport, represent the hardware (infrastructure & service delivery) of the health system that can undermine or strengthen the implementation of the co-created interventions and sustainability thereof. These require complementary processes within a broader health system-strengthening strategy to build resilient health systems.

Engaging communities in childhood immunization programming efforts presented both opportunities and challenges, as demonstrated by the project. The collective function of diverse actors in this approach, from caregivers at the community level and healthcare workers to representatives at the district and national levels, fostered a deep sense of community, inclusion, ownership of the process, and solutions [20, 21]. Collective engagement is crucial for addressing real immunization barriers and bringing about sustained change and action [38, 39].

The structured alignment with existing immunization strategies, such as involvement of REDREC in Mozambique [41] and the Mothercare programme in Malawi [42], is another opportunity that has potentially laid a strong foundation for the interventions' sustainable integration into broader immunization programmes. Involving the Ministry of Health is crucial for the approval of interventions and is a key facilitator for integration and scalability to other contexts. However, maintaining the long-term motivation of community actors and ensuring their continued commitment to immunization efforts can be challenging, especially without sustained external support. A collaborative approach to sustainability planning and ensuring ongoing engagement, resources, and support for these community actors is essential to the enduring success of immunization efforts.

### Key components essential for the approach’s success

**Fig. 1:**
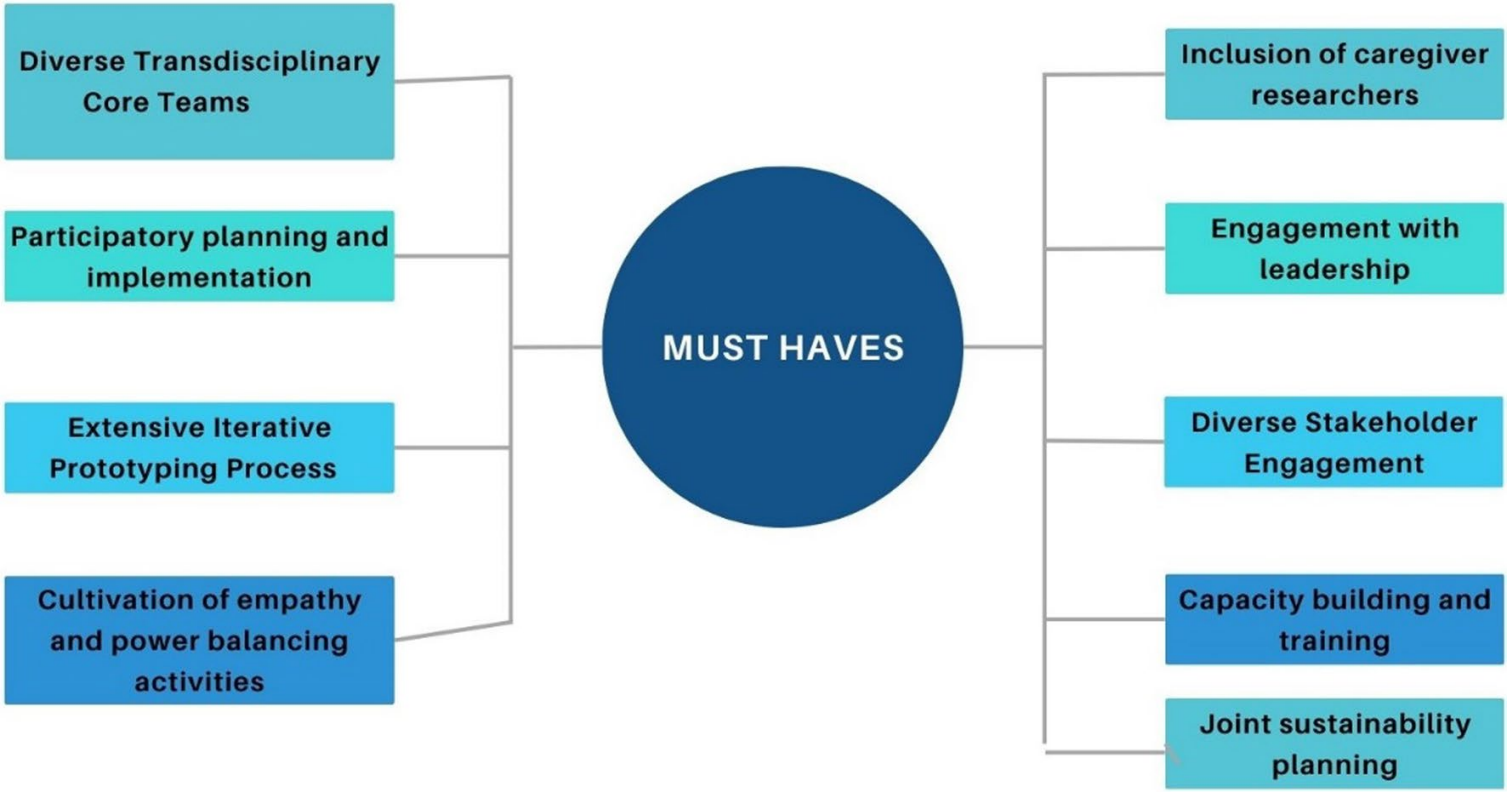
Foundational elements for the success of the approach

Figure 1 below summarizes the key components identified in our study, which are considered foundational elements essential in ensuring implementation success. These findings are consistent with Chen et al.'s [9] recommendations for five fundamentals applied to incorporate HCD strategies when implementing CBPR projects.

By prioritizing the above elements, similar initiatives can potentially replicate the success of the approach.

## Recommendations

To ensure a more comprehensive and inclusive approach to the CBPR/HCD approach, several recommendations can be considered. These include the development of simple manuals using accessible language, adapting methodologies to contextual realities, establishing early collaboration with the Ministry of Health, and reinforcement of policies that support the incorporation of community feedback and codesigning of solutions. Future research could also explore techniques such as Propensity Score Matching and Most Significant Change to strengthen the attribution and robustness of the process evaluation findings.

## Conclusion

Integrating CBPR and HCD into routine immunization programs has the potential to enhance community-responsive programming in LMICs, leading to effective and sustained changes. The ‘Let’s talk about vaccines’ approach provides key elements and factors for improving immunization initiatives. It offers insights into opportunities and challenges as a blueprint that can potentially be adapted to similar contexts for engaging communities in immunization efforts. The approach provides key components as practical strategies on how CBPR/HCD can be replicated in similar contexts to improve immunization outcomes. By incorporating community feedback and co-designing interventions that address specific needs, the CBPR and HCD approaches build trust, improve community engagement, and potentially improve immunization access and uptake, even in challenging settings.

## Contribution to literature

This process evaluation adds empirical evidence to support the complementary use of CBPR and HCD in childhood immunization efforts. This study addresses the gap in the literature regarding the co-creation of health interventions, particularly in the immunization space, where traditional methods have often overlooked the insights of caregivers and healthcare workers. By employing participatory methods, the ‘Let’s talk about vaccines’ approach demonstrates how local knowledge and community engagement can lead to tailored and more acceptable solutions to address immunization barriers. Additionally, the use of theoretical frameworks, such as RE-AIM and CFIR, provides a structured embedded process evaluation to capture the implementation process. This offers a model for future research.

## Data Availability

The data that support the findings of this study are available from the University of the Western Cape (UWC), but restrictions apply to the availability of these data and so are not publicly available. The data are, however, available from the authors upon reasonable request and with the permission of the institution.

## Appendix 1: Location, category and number of participants

**Table:**

Mozambique		
Location	Category of participant	Number
Gile	Caregiver researchers	2
	Caregivers	9
	Healthcare Workers responsible for EPI	2
	Community leadership	5
	District health officials	4
Namarroi	Caregiver researchers	
	Caregivers	3
	Healthcare Workers responsible for EPI	2
	Community leadership	6
	District health officials	4
Zambezia Province	Provincial health officials	3
VillageReach	Global and country staff	5

**Table:**

Malawi		
Location	Category	Number
Lilongwe	Caregiver researchers	1
	Caregivers	2
	Health Surveillance Assistants	4
	Community leadership	1
	Community volunteers	2
	District health officials	2
	Drama group representative	1
Mzimba North	Caregiver researchers	1
	Caregivers	2
	Health Surveillance Assistants	4
	Community leadership	1
	Community volunteers	2
	District health officials	2
	Drama group representative	1
	Mothercare Group representative	2
VillageReach	Country staff	3

## Appendix 2: HCD feedback surveys

Summary of responses from the HCD feedback survey in Mozambique at the prototype workshop.

Summary of responses from the HCD feedback survey with Lilongwe participants at the HCD ideation workshop.

Summary responses of the HCD feedback survey in Mzimba North at the HCD ideation workshop.

## Appendix 3: Alignment to the core CBPR principles in Mozambique and Malawi

**Table:**

CBPR Principle	Degree met	VillageReach approach and alignment
Recognizes community as a unit of identity	Substantial	The project established a shared sense of identity by fostering collaboration among caregivers, stakeholders in immunization, and those influencing immunization uptake to work together A detailed definition of community and setting (2 districts) established. VillageReach had a clearly defined population with the main target population as caregivers with children between 25 and 34 months and other intended users (health care workers) and stakeholders involved in childhood immunization programming (district and provincial health officials)
Builds on strengths and resources within the community	Substantial	In Mozambique, the research was driven by the provincial, district and community structures and relied on community and resources to identify participants In Malawi, the research relied on community and health structures, employing tools like health register books, tally sheets, and collaboration with community volunteers, HSAs, and community leaders to identify and track missing children and their caregivers
Facilitates collaborative, equitable involvement of all partners in all phases of the research through an empowering and power sharing process that attends to social inequalities	Substantial *but limited in the initial start of the project*	VillageReach *created a diverse study team* with complementary roles and included the intended target population/user as a caregiver researcher helped shape purpose and scope of the project to ensure more validity in the research approach and promote input/ buy-in of the findings among communities and provincial/district governments. Training and feedback processes on qualitative research and co-analysis were incorporated, promoting mutual learning. Virtual debriefs were frequently conducted, enhancing capacity building. However, caregiver researchers were not initially involved in conceptualization, i.e., IRB protocol development and development of initial tools
Promotes co-learning and capacity building among all partners	Substantial	VillageReach created opportunities for the exchange of knowledge, experience, and training through orientation training, virtual data collection debrief sessions on data collection, training on CBPR, *caregiver researcher training and support*, etc. The joint knowledge and experiences were applied sufficiently to the planning (joint development of proposal and tools) and implementation of the research project there was evidence of mutual knowledge transfer, with the study and HCD creating opportunities to understand context and barriers. This resulted in increased capacity among different stakeholders in research and under two immunization
Integrate and achieve a balance between data generation and intervention for the mutual benefit of all partners	Substantial	VillageReach satisfactorily used participatory tools (photovoice, telegram) to generate data and HCD methods to validate findings and generate community driven solutions based on the data and representative participants contributions A clear process was followed with all partners and caregivers, from data collection on barriers to solution generation and the commencement of implementing the solution components
Focus on the local relevance of public health problems and on ecological perspectives that attend to multiple determinants of health	Substantial* to a lesser extent*	VillageReach used participatory data collection methods with caregiver and health care workers to identify relevant determinants of childhood immunization access and uptake in order understand drivers of under two routine immunization (RI) dropout from the perspectives of caregivers The approach notably tackled locally relevant barriers faced by caregivers but showed limitations in addressing the multiple determinants of low under two immunization access and uptake, primarily due to funding constraints
Involve systems development in a cyclical and iterative process	Substantial	VillageReach undertook to commit to a time intensive process to generate data through CBPR, presenting findings to participants, consultation with health officials, other relevant partners and health promotion expertise, a situational analysis and an iterative HCD process (ideation, prototype) to generate solutions The approach incorporated several steps to develop solutions, consult stakeholders on findings, and create tailored solutions
Disseminate results to all partners and involve them in the wider dissemination of results; and	Substantial	VillageReach established a significant number of opportunities to present the findings of the study with the participants at the health facility level, district, and provincial health leadership, MISAU and met with other partners in the immunization space. VillageReach also facilitated co-authorship with those involved to ensure that their contributions are recognized through peer-reviewed authorship. The CBPR results were published in BMJ Open – “*Determinants of immunisation dropout among children under the age of 2 in Zambézia province, Mozambique: a community-based participatory research study using Photovoice*)” *Using community-based, participatory qualitative research to identify determinants of routine vaccination drop-out for children under 2 in Lilongwe and Mzimba North Districts, Malawi* Results were disseminated at various stages to all stakeholders
Involve a long-term process and commitment to sustainability	*To a limited degree but with initial planning and consideration*	VillageReach established a monitoring system of solution implementation; however, it is too early to determine sustainability aspects or securing grants to expand the program, but initial sustainability planning is underway Collaborative teams and mutual relations were established at various stages, suggesting potential continuity beyond the research project

## Appendix 4: Supporting quotations on adoption

**Table:**

CFIR Construct	Supporting Quotations
Intervention source	How participants identified barriers and interventions locally and the feasibility of the solutions: “The study arrived in the district and we did the interviews, we brought the problems. Each district realized what the problems were, and we created solutions, the district welcomed these solutions and now we are waiting for a change of scenery. Meanwhile, those basic solutions (low expensive) are being implemented at the local level.” (Health official, Mozambique) “Yes, from the solutions we identified if they get implemented, they have the capacity to address the problems or reduce them.” (SHSA, Malawi)
Evidence strength and quality	Health officials, in both Malawi and Mozambique, reflected on the satisfaction with the alignment of results with community and stakeholders’ perspectives: “These findings [CBPR study findings] were shared in a meeting [referring to HCD workshop] like we would organize a meeting where we shared. We also discussed about these things where they are saying because of lack of support, lack of male involvement let’s say … there were shared in a meeting.” “… and the results were also satisfactory because after finishing the data collection, we had a workshop that involved the health facilities, the community leaders, and some participants. Because after we gather the information [immunization barriers] we have those preliminary results,….” (Health official, Mozambique)
Relative advantage	In Mozambique a health official emphasized: “The project had an important component of involving mothers and/or caregivers with children under 2 years old, …the mothers who knew that they had children that they should vaccinate, because if it involved, for example, men OR women who did not have these children, at some point they might not understand in that period what that means, so this was a great advantage.” (Health official, Mozambique) Similarly, in Malawi, an SHSA underscored the benefits of the new approach in improving vaccination records and reducing instances of missing vaccinations: “The method that was before, children were missing a lot, not knowing which vaccine the kid has received, …that’s why the new method…has helped us.” (SHSA, Malawi)
Complexity	A health official and HSA noted: “It’s not that much complicated… But in terms of moving on having it [solution components] being used, I think it’s not that complicated but the start-up is what is giving out some complications we really need to agree on what it is that we are really going to disseminate.” (Health official, Malawi) “The decisions that were made are relevant, but they cannot end all the problems in our area. Like on the issue of shelter, it can help, but the issue of transport still remains a challenge when it comes to getting the vaccine from the health center to the outreach clinic. These decisions they have the possibility of reducing the challenges but not ending them.” (HSA, Malawi)
Effective networks and communication	Participants emphasized the impact of these efforts: “This Bate-Papo project, in fact, as the name says, helped positively in the reduction of absent children in health facilities, because after all these meetings, since they included all community leaders, religious leaders, APSs, traditional medicine practitioners, the Government itself, the district, the locality, they realized the consequences that can arise if the children do not finish the vaccine.” (Health official, Mozambique) “Now the specific teams we have established are drama groups, community volunteers, the ones that will assist to give messages to the villages…So, we have these caregivers that understand the concept of immunization and these are the ones that we give them the task to teach others in the community like PR.” (Health official, Malawi)
	In Mozambique, a healthcare worker described how the HCD process involved discussing difficulties and proposing interventions collectively: “Normally we did HCD where we put the difficulties and the possible answers, the possible solutions. For example, in the case of long distances, we talked about the need for the family to help and not only that, we talked about mobile brigades and the need to associate the formation of these brigades with the involvement of the community (from the community leaders for mobilization). So, that was the consensus.” (Health care worker, Mozambique) Similarly, in Malawi, a caregiver researcher emphasized the collaborative nature of decision-making, where participants worked in groups to identify and prioritize solutions: “Yes, people were working in groups, then in those groups, they were agreeing, they would discuss, ok how best do you think this can be dealt, how then they had to come up with as this group this is what we have agreed and then they come up with three points in each group and then out of those the whole team called the, it’s what participants voted against who come at the top priorities.” (Caregiver Researcher, Malawi)
Implementation climate	In response to the question “So, where the solution is now, do you see that your contribution is evident in that solution?”; participants commented: “I believe so, because at the beginning of the workshop, throughout and until now, when I see the points raised there, I see some points that I reported, for example, the issue of distance.” (Healthcare worker, Mozambique) “…, one of the examples was to form the APSs, within the scope of REDREC, and it has already happened. Another is to form the APSs to carry out the mobilization and it is already happening. And besides that, there are the regular meetings that we are having with the community leaders to guarantee the mobilization …My contributions were recognized,” (Health official, Mozambique) Additionally, a caregiver from Malawi highlighted the inclusive nature of the decision-making process, where everyone was given an opportunity to contribute: "Everyone was given a chance, and it was your choice not to speak but the opportunity was given to everyone.” (Caregiver, Malawi)
	A health official emphasized the potential for integration, stating: “See, we cannot talk about integration without the mobilization and the Bate-Papo project is strong in the community mobilization component. Then it could be integrated there with more and more capacity building for the focal points, APSs, even the members of the health committees, thus entering into the vaccination program. Later it can be integrated into logistics.” (Health Official, Mozambique)
External policies and incentives	In Mozambique, a health official noted the project's alignment with provincial objectives, particularly with regard to the Expanded Program on Immunization: “The Bate-Papo project identifies with the health province objectives, especially with the EPI. And at the level of each district, it is welcomed because it is perceived that the Bate-Papo helps the district to improve its indicators at the program level and reduce the number of children with zero dose and increase the coverage of fully vaccinated children." (Health official, Mozambique) Similarly, in Malawi, a health official highlighted the project's origins from within the community, indicating a sense of ownership and investment in its success: So, for us, I feel like we cannot have much resistance because it is something which came from us and we looked into in some area, district, community behavior and some approaches which we can take up.” (Health official, Malawi)
